# *Coleophora nepetellae* Baldizzone & Nel, a new species of the *C. lixella* group (Lepidoptera, Coleophoridae) from France and Italy

**DOI:** 10.3897/zookeys.459.7983

**Published:** 2014-12-02

**Authors:** Giorgio Baldizzone, Jacques Nel, Jean-François Landry

**Affiliations:** 1 Via Manzoni, 24, I-14100 Asti, Italy; 2 78, Avenue Fernand Gassion, F-13600 La Ciotat, France; 3 Agriculture and Agri-Food Canada, C.E.F., 960 Carling Avenue, Ottawa, Ontario K1A 0C6, Canada

**Keywords:** *Coleophora caucasica*, *Coleophora lixella*, *Coleophora malatiella*, *Coleophora nevadella*, *Coleophora ornatipennella*, *Coleophora samarensis*, *Coleophora tricolor*, DNA barcodes, genitalia, *Nepeta*

## Abstract

*Coleophora nepetellae* Baldizzone & Nel, **sp. n.** is described from the southern Alps (Italy and France). It belongs to the *Coleophora lixella* species group. Its host plants are *Nepeta nepetella* L. (Lamiaceae) and an unidentified Poaceae. The fifth instar larva, its case, the adult habitus, and genitalia are illustrated. The species is compared to *Coleophora nevadella* Baldizzone, 1985, here newly confirmed from France and whose larvae feed on *Nepeta latifolia* DC. in the Eastern Pyrénées. DNA barcodes are shown to be distinct and congruent with morphological differences among species of the *lixella* group. Barcodes revealed that *Coleophora tricolor* Walsingham, 1889, formerly known only from Great Britain, is also present in France and Greece.

## Abstract (fr)

*Coleophora nepetellae* Baldizzone & Nel, **sp. n.** du groupe de *Coleophora lixella* Zeller, 1849., est décrite des Alpes méridionales (Italie et France). La plante-hôte de ponte est la Lamiacée *Nepeta nepetella* L. et celle de la larve à maturité est une espèce non-identifiée de Poaceae. La chenille L5 et son fourreau, l’habitus et les genitalia mâles et femelles sont figurés. L’espèce est comparée à *Coleophora nevadella* Baldizzone, 1985, espèce ici confirmée pour la France où elle est inféodée à *Nepeta latifolia* DC. dans les Pyrénées-Orientales. Enfin, la validité de cette nouvelle espèce est confirmée par l’étude des codes-barres ADN du groupe *lixella*. Les codes-barres ont aussi révélé la présence de *Coleophora tricolor* Walsingham, 1889 en France et en Grèce alors que l’espèce n’était auparavant connue que de la Grande-Bretagne.

## Introduction

The *Coleophora lixella* group is currently composed of the following seven species: *Coleophora ornatipennella* (Hübner, 1796), *Coleophora lixella* Zeller, 1849, *Coleophora caucasica* Stainton, 1867, *Coleophora tricolor* Walsingham, 1899, *Coleophora malatiella* Toll, 1952, *Coleophora nevadella* Baldizzone, 1985, and *Coleophora samarensis* (Anikin, 2001). The group composition was initially circumscribed by Toll (1952), and subsequently expanded by Baldizzone (1985) and Anikin (2001) to include their newly described species.

The group is defined by the following combination of characters: apically falcate forewings; antennal scape with a long tuft, base of the flagellum thickened with spreading scales; very elongate, narrow tegumen with almost straight sides and abruptly widened and angled pedunculi; elongate and upcurved cucullus; very elongate vesica about twice the length of the phallus; very long phallic appendix with 10–20 coils; sterigma with two elongate, digitiform lateral bars; colliculum very slender and as long or longer than S8; spinulate section of ductus bursae with one very long coil; papillae anales thickened and melanized; larva feeding on two different host plants, the florets or seeds of a Lamiaceae in its early stages, switching to mining leaves of Poaceae in its late stages, making a different case on each host. It remains undetermined whether these features are apomorphic or have phylogenetic value, but members of the group share a distinctive external aspect and male and female genitalia, and the switch from dicot to monocot hosts during larval development is unique.

Emmet (1996, p. 128, 276) seems to be the sole author to have also included *Coleophora ochrea* (Haworth, 1928) in the *lixella* group. However, his reason for doing so was not explicitely stated and was probably based on similarities that he mentioned in his diagnosis of the group, notably the tubular phallus, short sacculus, the antennal scape with a long tuft, the base of the flagellum thickened with spreading scales, and the falcate forewings. None of these characters is unique to the group. *Coleophora ochrea* differs in several male and female genitalia features from species of the *lixella* group, notably the differently shaped, shorter tegumen, short and spatulate cucullus, lack of lateral bars on the sterigma, colliculum shorter than S8, and the short and straight spinulate section of the ductus bursae. The case-making and feeding habits of its larva are different as well: unlike members of the *lixella* group, *Coleophora ochrea* uses a single host plant during its development and enlarges its case in sections as it grows. Because of these differences, we exclude *Coleophora ochrea* from the *lixella* group, although its affinities remain undetermined. The recent phylogenetic (molecular) framework for Coleophoridae (Bauer et al. 2012) did not analyze *Coleophora ochrea*.

The most widespread species in the *lixella* group is *Coleophora ornatipennella*, which is distributed over most of Europe and extends in Asia from Turkey to Siberia and China; *Coleophora tricolor* was known only from Great Britain but is here reported from continental Europe; *Coleophora malatiella* is known from Romania, Turkey, Ukraine, and Iran; *Coleophora nevadella* was formerly known only from Spain but its presence in the western Pyrénées region of France is established in the present work (see below); *Coleophora samarensis* is known from southern Russia and from Georgia to the Ukraine; and *Coleophora caucasica* is known from Georgia (where the type locality is), Armenia, and Turkey (Baldizzone et al. 2006). Details of the larval life history are provided in Emmet (1996) for the two British species, *Coleophora lixella* and *Coleophora tricolor*.

In recent years DNA barcoding of many specimens has led to a re-assessment of species occurrences (Landry et al. 2013) as well as revealed the presence of additional, undescribed species. Among the recently detected undescribed species is the one we describe as new in the present work. In 2001, JN had already recorded it in his Atlas under entry no 140b as “*Coleophora lixella (Eupista)* cf. *malatiella* Toll, 1952”, in addition to noting its close relationship to specimens of *Coleophora nevadella*. In 2012, GB found in the Varaita Valley (Cottian Alps, Piedmont) of northern Italy a large population of a *Coleophora* associated with *Nepeta nepetella*: the adults were bigger and looked different from typical *Coleophora lixella*. Examination of the genitalia confirmed that it was an undescribed species closely related to *Coleophora nevadella*, and that it matched species 140b reported in Nel (2001).

## Taxonomy

### Coleophora nepetellae

Taxon classificationAnimaliaLepidopteraColeophoridae

Baldizzone & Nel sp. n.

http://zoobank.org/8F1DE90F-4535-49E5-945E-1BBCFA933E3A

Barcode Index Number: BOLD:AAI9227

#### Type material.

Holotype ♂ (genitalia slide Bldz 15711): [**Italy**] “PIEMONTE |V.[alle] Varaita | Pontechianale (CN) | Grangia del Rio 2000 m | 30-VII-2012 | G. Baldizzone *leg.*”; “Database # | CNCLEP | 00110051”; “Barcode of Life Project | Leg(s) removed | DNA extracted” [blue]. In coll. Baldizzone, Asti.

Paratypes: **Italy:** 14 ♂, 12 ♀, Piemonte, same locality and date as holotype, coll. Baldizzone, barcoded specimens # CNCLEP00110052–CNCLEP00110060; 14 ♂♂ (genitalia slide Bldz 15535), 12 ♀ (genitalia slide Bldz 15536, 15712), *ibidem*, 2-VIII-2012, coll. Baldizzone; 4 ♂, 8 ♀, *ibidem*, 22.VII.2013, coll. Baldizzone; 1 ♂, *ibidem*, 2.VIII.1986, G. Bassi leg., coll. Bassi.

**France:** 1 ♀, Alpes-Maritimes, Le Pra sur Tinée, 1700 m, 44.3227°N, 6.8849°E, ex *Nepeta nepetella*, 23.VII.2000, J. Nel *leg.*, coll. J. Nel, La Ciotat; 1 ♀, Alpes Maritimes, Roubion, 44.093°N, 7.0511°E, 1630 m, 9.VII.2011, Th. Varenne *leg.*, coll. Th. Varenne, Nice; 1 ♂, Alpes-Maritimes, Tende, col de Tende, 44.15°N, 7.5667°E, 1830 m, 21.VII.1995, Th. Varenne *leg.*, coll. Th. Varenne; 1 ♀, Alpes-Maritimes, Casterino, 44.0986°N, 7.5059°E, 2000 m, 13.VIII.2013, ex *Nepeta nepetella*, J. Nel *leg.*, coll. J. Nel; 1 ♂, Alpes-de-Haute-Provence, St-Ours near Meyronnes, 44.4805°N, 6.8086°E, 15.VII.2005, 1800 m, *leg.* Jacques Nel, specimen # CNCLEP00033958, genitalia slide MIC 6834, barcoded, Canadian National Collection, Ottawa; 1 ♀, Alpes-de-Haute-Provence, 2 km E Meyronnes, 44.4711°N, 6.8086°E, 1575 m, 2.VII.2005, *leg.* C & FK Gielis, specimen # CNCLEP00029208, genitalia slide MIC 6835, barcoded, coll. H. van der Wolf, Netherlands.

#### Diagnosis.

*Coleophora nepetellae* is a relatively large *Coleophora*, whose forewings are predominantly yellow with fine silvery striae. It belongs to the *Coleophora lixella* species group and is most similar to *Coleophora nevadella*, both externally and in genitalia. The latter species was formerly known only from Spain (Vives Moreno 1991) but is here reported from France for the first time based on material collected by JN (see below).

Externally the adult of *Coleophora nevadella* from the Sierra Nevada (the type locality in Spain) (Fig. 2) is on average smaller than that of *Coleophora nepetellae*, its forewings are devoid of brown scales, and the silvery striae are inconspicuous or nearly absent. In male genitalia (Figs 6, 11), *Coleophora nevadella* has the valvula smaller with its outer margin more oblique and its ventral margin barely reaches the dorsal edge of the sacculus, whereas in *Coleophora nepetellae* the ventral margin of the valvula is extended nearly to the ventral edge of the sacculus. The apex of sacculus is more prominently bulged in *Coleophora nepetellae* than in *Coleophora nevadella*.

In female genitalia, *Coleophora nepetellae* is easily distinguished from *Coleophora nevadella* by the following differences: in *Coleophora nevadella* the colliculum is more elongate and has two long and thin lateral bars that are extended nearly to the distal margin of the sterigma (Fig. 12); these bars are very short in *Coleophora nepetellae* (Fig. 13); in *Coleophora nevadella* the median lamina in the ostium bursae is long, thin, and extended posterad of the ostium whereas in *Coleophora nepetellae* it is more robust, irregularly delineated and with sclerotized lateral extensions; finally the paired longitudinal bars of the sterigma are narrow with the inner margins smooth in *Coleophora nevadella*, whereas they are rough-edged with small, spinulose protuberances in *Coleophora nepetellae*.

#### Description.

*Adult*. Wingspan 22–26 mm. Head white shaded with yellow on vertex. Antenna with scape cream yellow, with thick tuft of erect, concolorous scales; flagellum white annulated with pale ochreous yellow, nearly indistinctly so on upper surface, dark brown on lower surface; basal third with elongate cream-coloured scales. Labial palp white, third article about as long as second; second article with long tuft of erect scales on ventral surface. Thorax white with a median cream yellow line; tegula cream with thin white border.

Forewing with apex falcate, curvature variable; ground colour cream yellow, paler in dorsal half, costal half slightly darker from scattering of brown scales, mainly in area between median line and costa; apical portion with 4–5 short, oblique silvery striae; one fine silvery stria in subcostal area near base, widening to quarter of wing and lined with brown on costal side; second silvery stria in median area from basal third to margin; third silvery stria along anal fold and interrupted before margin; fourth silvery stria along dorsal margin, very short and inconspicuous. Fringe dark cream-coloured along costal margin and around falcate apex; on dorsal margin, fringe pale grey with a line of pale cream basally. Hindwing grey, sometimes with brownish hue, fringe coloured as in forewing. Abdomen pale dirty white.

*Abdominal apodemes* (Fig. 8): Latero-anterior bars about twice as long as latero-posterior ones. Transverse bar long, proximal edge straight and thin, distal edge slightly convex around tergal sclerites. Tergal sclerites covered with conical spines, about 5–6× longer than wide (on T3).

*Male genitalia* (Figs 6, 7, 11): Gnathos knob large, globose. Tegumen narrow, elongate, pedunculi slightly outwardly flared. Transtilla thin, linear. Valvula wide with rounded ventral margin. Cucullus large, markedly sclerotized, wider basally, obliquely oriented, dorsal side slightly convex and apex rounded. Phallotheca elongate-conical, ventral portion less sclerotized. Cornuti (Fig. 7) thin, tightly arranged in elongate bundle.

*Female genitalia* (Figs 9, 13): Papillae anales markedly sclerotized, elongate. Posterior apophysis twice as long as anterior one. Sterigma conical laterally with pair of longitudinal sclerotized, outcurved bars, curvature more pronounced on outer side, distal portion of bars parallel to each other. Ostium bursae calix-shaped. Widest distal section of colliculum with small conical spines, proximal section narrowed with lateral edges more sclerotized, median lamina extended from smooth, looped section of ductus bursae to widened portion of colliculum. Ductus bursae lined with short conical spinules from anterior end of colliculum to first loop (about 4× length of sterigma); second loop without spinules (about 2× length of sterigma); anteriormost section of ductus transparent, without ornamentation. Corpus bursae ovoid with distal half tapered, signum leaf-like.

**Figure 1.**
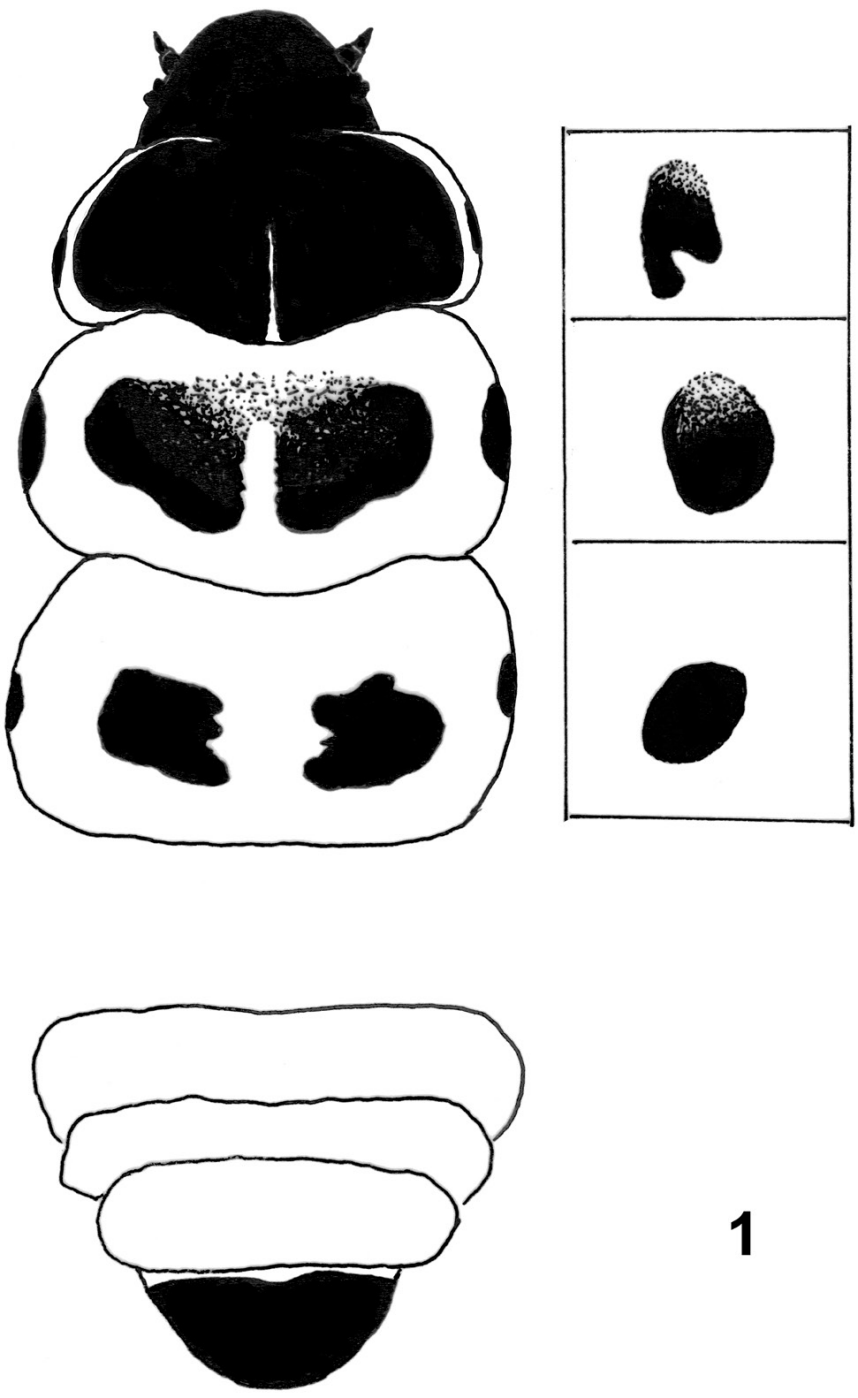
*Coleophora nepetellae*, schematic illustration of 5th larval instar.

*L5 larva* (Fig. 1): Length 9 mm. Body brown with faint brown dorsal line interrupted at segment junctions. Head shiny black. Thoracic shields shiny black; prothoracic shield wide, very finely cracked along median axis from middle to posterior edge; mesothoracic shield made up of two wide figs irregular in shape, separated by a median gap except thinly and narrowly joined anteriorly; metathoracic shield made up of two smaller figs separated by a gap, irregular in shape with denticulate inner edges. Spiracular sclerites shiny black, present on all thoracic segments: oblong on prothorax, oval on mesothorax and metathorax, largest in size on mesothorax. Thoracic legs entirely shiny black. Prolegs on A3–A6 with 5–7 crochets in two uniordinal rows. Anal fig shiny black. Anal proleg half-moon-shaped, each with 15–18 crochets. (Description based on larva from France, Alpes-de-Haute-Provence, Montagne de Lure, 1519 m, 31.V.2013, found on grass beside *Nepeta nepetella*, J. Nel *leg.*).

#### Derivation of specific epithet.

The species epithet is derived from the species name of its larval host, *Nepeta nepetella*.

#### Biology.

The host plant of *Coleophora nepetellae* is *Nepeta nepetella* L. (Lamiaceae). This plant has narrow, dentate, whitish green leaves, and large white, hairy corollas with the pistils extended beyond the chalices. This is a mountain plant that occurs throughout the southern Alps, in France mainly in the most sun-exposed parts of the foothills, between 1000 and 2000 m in elevation, along roads, at the base of screes, or near sheepfolds. It blooms between mid-June and late August, depending on elevation and exposure. *Nepeta nepetella* is the initial food plant from which the larva makes its first case. The second host plant which serves to construct the final case is a unidentified Poaceae.

Oviposition and larval development between late summer and overwintering was not observed directly but it can be inferred with much certainty that the eggs are deposited on or in the chalices, and that the young larvae build a case from a seed, as do all the other species of the *lixella* group for which the biology is known. Overwintering probably takes place on the ground or in the litter. In the spring, it was observed that the young larva lives in a case made from a hollowed-out piece of grass. Grasses used for case-making are species with broad leaves that grow in close proximity to *Nepeta* plants which become the larval host. This habit of using different host plants before and after overwintering is exceptional among leaf-mining Lepidoptera and occurs among all the species of the *lixella* group for which the biology is known. The case is not enlarged during larval development (as in *Coleophora ornatipennella*, for example) but is abandoned for a new, larger one at each instar. The final case (Fig. 5) is constructed from a piece of mined grass leaf which is hollowed out. It is 13–15 mm long, about 3 mm at its widest girth, straw-coloured, slightly darkened, with longitudinal ridges made by the veins from the leaf used in its construction; the oral opening is rounded, at 30°; the anal end has the terminal 3 mm dorso-ventrally flattened; the median portion is slightly broader, fusiform.

#### Phenology.

The species has one generation per year, with the adults emerging between July 20 and the first week of August in the Valle Varaita in Italy. In France at higher elevations adult emergence extends into the middle of August. In all locations adult flight coincides with the flowering of the host plant, *Nepeta nepetella*. The adults fly in bright sunshine, especially during the afternoon and take short flights among the flowering stems. The new species coexists with *Coleophora lixella* which flies among Thymus cf. serpyllum L. (Lamiaceae), its host plant.

#### Type locality.

Italy, Piemonte, Valle Varaita, Pontechianale, Grangia del Rio, 2000 m, 44.6625°N, 6.9939°E. The Grangia del Rio is a side valley of the Valle Varaita through which runs a tributary of the Varaita River. It is situated in the Cottian group of the Western Alps in northwestern Italy. The host plant, *Nepeta nepetella*, from which type material was obtained grows there within 30–40 feet of a pastoral trail and at the base of a rock slide.

#### Geographical distribution.

In Italy the species is known only from the type locality in the Piemont Region. In France, it is recorded from the Alpes-Maritimes, Upper Var, Alpes-de-Haute-Provence, Hautes-Alpes, Isère, the Drôme, and further to the west from the Vaucluse where it is common on the slopes of Mont Ventoux wherever *Nepeta nepetella* grows.

##### Records of *Coleophora nevadella* from France

In his Atlas of Coleophoridae of France, Nel (2001) recorded a species of the *lixella* group from the Eastern Pyrénées (Cerdagne area). He labelled it “*Coleophora lixella* (*Eupista*) cf. *nevadella* Baldizzone, 1985” (under his entry no 140c) and indicated that the identification was tentative. *Coleophora nevadella* was formerly known only from Spain (Vives Moreno 1991). Nel reported finding adults among *Nepeta latifolia* DC., a Lamiaceae distributed in the Iberian Peninsula which reaches its northern limit in southwestern France. We confirm here that *Coleophora nevadella* is indeed the species tentatively reported from France by Nel (2001). The adults occur in July on blooming *Nepeta latifolia* plants, which is the likely oviposition host plant.

**Record details.** 3 ♂, 2 ♀,: France, Pyrénées-Orientales, Mont-Louis, route D10 to Sauto 11.VII.1990, imagos on *Nepeta latifolia*, J. Nel leg.; 2 ♂, ditto, 28.VII.1993. 6 ♂, Pyrénées-Orientales, Védrignans, 1400 m, 16.VII.1992, imagos on *Nepeta latifolia*, J. Nel leg.; 1 ♀, ditto, 30.VII.1993 (Jacques Nel Collection and Tyroler Landemuseen, Innsbruck). 1 ♂, Porté-Puymorens, vallon de Passet, 1650 m, 17.VII.2004, T Varenne leg. (Thierry Varenne Collection, Nice).

**Figures 2–5.**
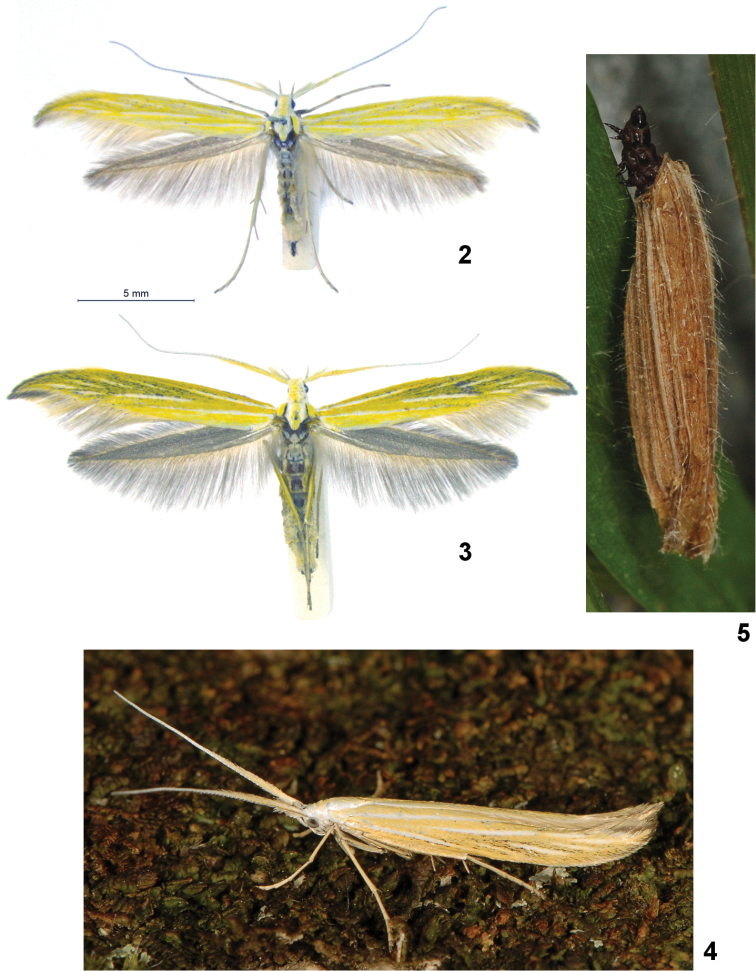
Adults and larval case of *Coleophora nepetellae* and *Coleophora nevadella*. **2** *Coleophora nevadella*, female: Spain, Sierra Nevada, Camino de la Veleta, 1600 m, 19.VII.1985, G. Baldizzone and E. Traugott-Olsen leg. (coll. Baldizzone) **3** *Coleophora nepetellae*, female paratype: Italy, Piemonte, Valle Varaita, Pontechianale, Grangia del Rio, 2000 m, 22.VII.2013, G. Baldizzone leg., (coll. Baldizzone) **4** *Coleophora nepetellae*, female paratype: France, Alpes Maritimes, Roubion, 1630 m, 09.VII.2011, Th. Varenne leg. (coll. Th. Varenne) **5** *Coleophora nepetellae*, larva in it case: France, Alpes-de-Haute-Provence, Montagne de Lure, 1519 m, 31.V.2013, on grass near *Nepeta nepetella*, J. Nel leg. (photo © Th. Varenne).

**Figures 6–9.**
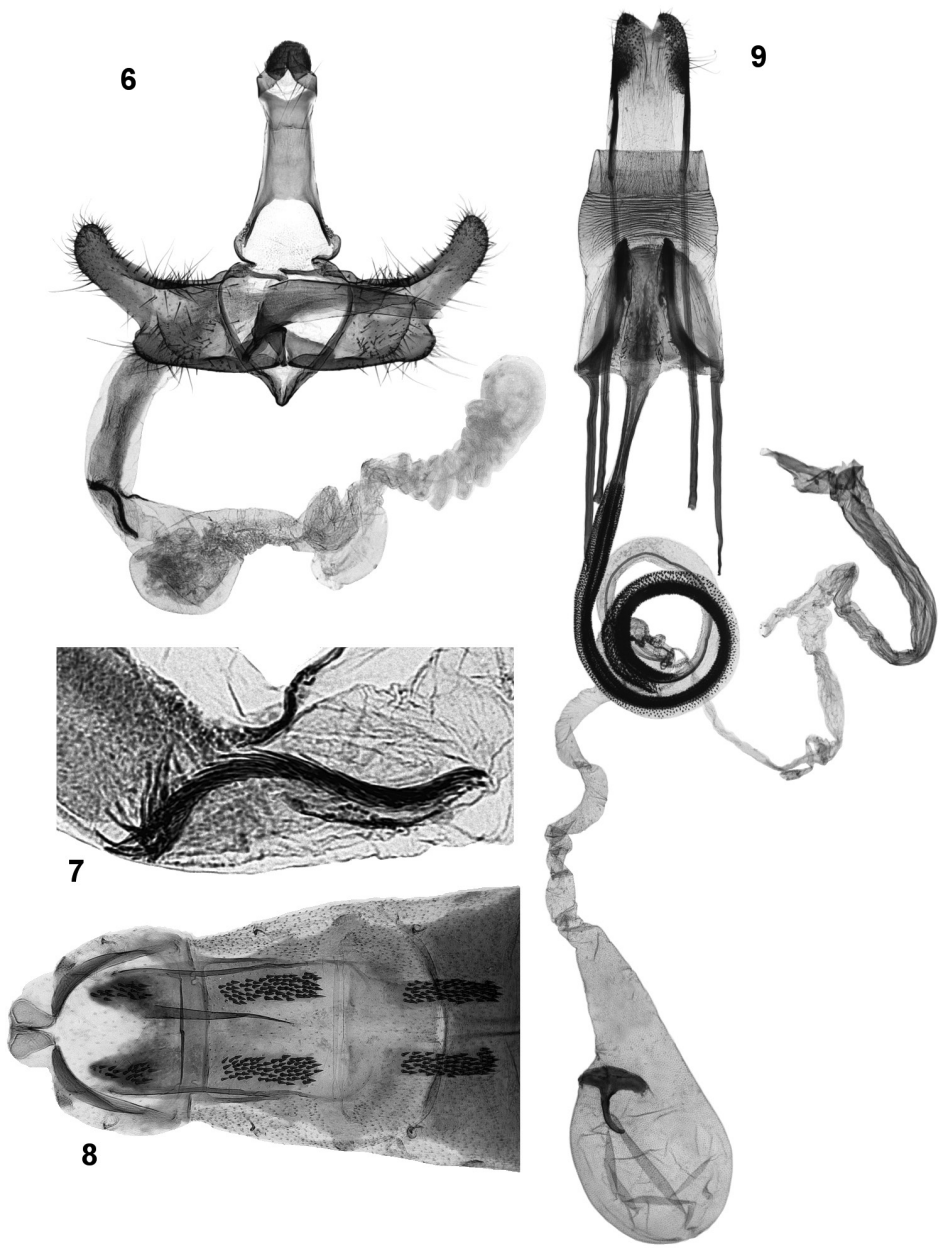
Genitalia of *Coleophora nepetellae*. **6** male genitalia (genitalia prep. Bldz 15535) **7** male genitalia, closeup of cornuti (genitalia prep. Bldz 15711, holotype) **8** Male abdominal segments 1–3 (genitalia prep. Bldz 15535) **9** Female genitalia (genitalia prep. Bldz 15536).

**Figures 10–13.**
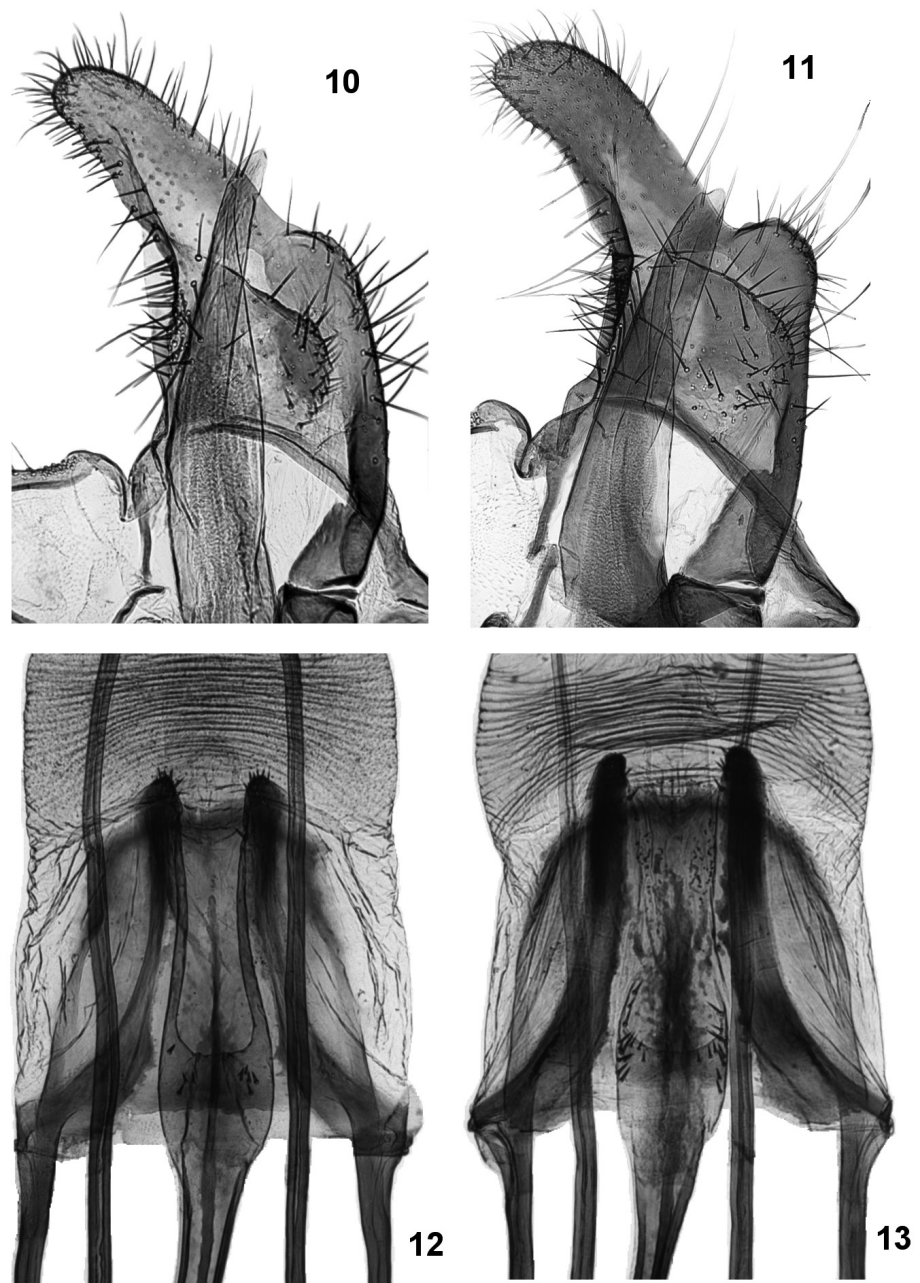
Genitalia of *Coleophora nepetellae* and *Coleophora nevadella*. **10** *Coleophora nevadella*, male genitalia, closeup of valva and distal portion of phallotheca (genitalia prep. Bldz 6162 – paratype): Spain, Granada, Sierra Nevada, 1000 m, Puerta de la Ragua, 20.VII.1969, K. Sattler & D.J. Carter (coll. BMNH) **11** *Coleophora nepetellae* male genitalia, closeup of valva and distal portion of phallotheca (genitalia prep. Bldz 15535) **12** *Coleophora nevadella*: female genitalia, detail of sterigma and colliculum (genitalia prep. Bldz 15713): Spain, Sierra Nevada, Camino de la Veleta, 2050 m, 22.VII.1985, G. Baldizzone and E. Traugott-Olsen (coll. Baldizzone) **13** *Coleophora nepetellae* female genitalia, detail of sterigma and colliculum (genitalia prep. Bldz 15712).

## DNA barcode analysis

Tissue samples (dried legs) were shipped to the Canadian Centre for DNA Barcoding in Guelph for DNA extraction, amplification, and sequence analysis. Laboratory protocols at this facility have been optimized, and the current iteration can be accessed at http://www.ccdb.ca. In short, a small tissue sample is lysed and genomic DNA extracted using an automated, silica-based method; the COI barcode region is amplified via PCR using one or more primer sets (Hebert et al. 2013) and successful amplicons are then bi-directionally sequenced (deWaard et al. 2008). The resultant sequences, along with the voucher data, images, and trace files, are deposited in the Barcode of Life Data Systems (BOLD) (Ratnasingham and Hebert 2007; www.barcodinglife.org), with sequences > 600bp subsequently deposited in GenBank. Sequences longer than 100 bp were included in the analysis.

Barcoding efforts included the holotype and 11 paratypes of the new species as well as representatives of several species of the *lixella* group. Several specimens of this species group that had already been barcoded independently of the present work as part of the Lepidoptera barcoding campaigns were also selected for the comparative analysis.

The Barcode Identification Numbers (BINs) (Ratnasingham and Hebert 2013) in BOLD are used as registry designations for barcode clusters. Neighbor-joining trees and genetic distances were calculated with MEGA 5.05 (Tamura et al. 2011) using the Kimura two-parameter (K2P) model of base substitution (Kimura 1980). Details of the barcoded specimens and their photographs are available through the following dataset (http://dx.doi.org/10.5883/DS-CNEPETA). The same DOI provides access to the sequence records, trace files, and primer sequences used for PCR amplification, together with GenBank accession numbers.

### DNA barcode results (Table 1, Fig. 14).

Eighty-seven specimens were successfully sequenced, resulting in a 658 bp, full-length barcode fragment for 63 specimens, and fragments of more than 600 bp for a further 17 specimens; two sequences longer than 500 bp, and five sequences shorter than 400 bp were also included in the analysis, whereas sequencing of 11 specimens failed. Only one of the analyzed species, *Coleophora nevadella*, failed to yield any sequence, as did two of the *Coleophora nepetellae* paratypes. Failure to obtain *Coleophora nevadella* sequences (four specimens were processed) was disappointing because this is the species deemed morphologically most similar to *Coleophora nepetellae*, with which it is compared in the diagnosis above.

Barcodes from the holotype and 11 paratypes of *Coleophora nepetellae* were obtained. Six paratypes yielded full barcodes whereas five yielded sequences between 605–639 bp; the holotype barcode was 622 bp with two ambiguous positions. Barcodes of *Coleophora nepetellae* were compared to barcodes from 12 other BINs representing at least nine distinct or putative species in the *lixella* group. Inter-group distances ranged from 1.4 to 10.2%, with an average of 7.4%. *Coleophora nepetellae* is markedly divergent from all other *lixella*-group clusters, with distances ranging from 3.9 to 9.6%, with *Coleophora samarensis* the closest species. The intraspecific distance was low and varied from 0.1–0.8%, with an average of 0.3%. Despite the lack of *Coleophora nevadella* sequences, the wide barcode gaps observed among the established, morphologically distinct species of the *lixella* group strongly suggest that that species would also show marked barcode divergence from *Coleophora nepetellae* and others in the group.

**Figure 14.**
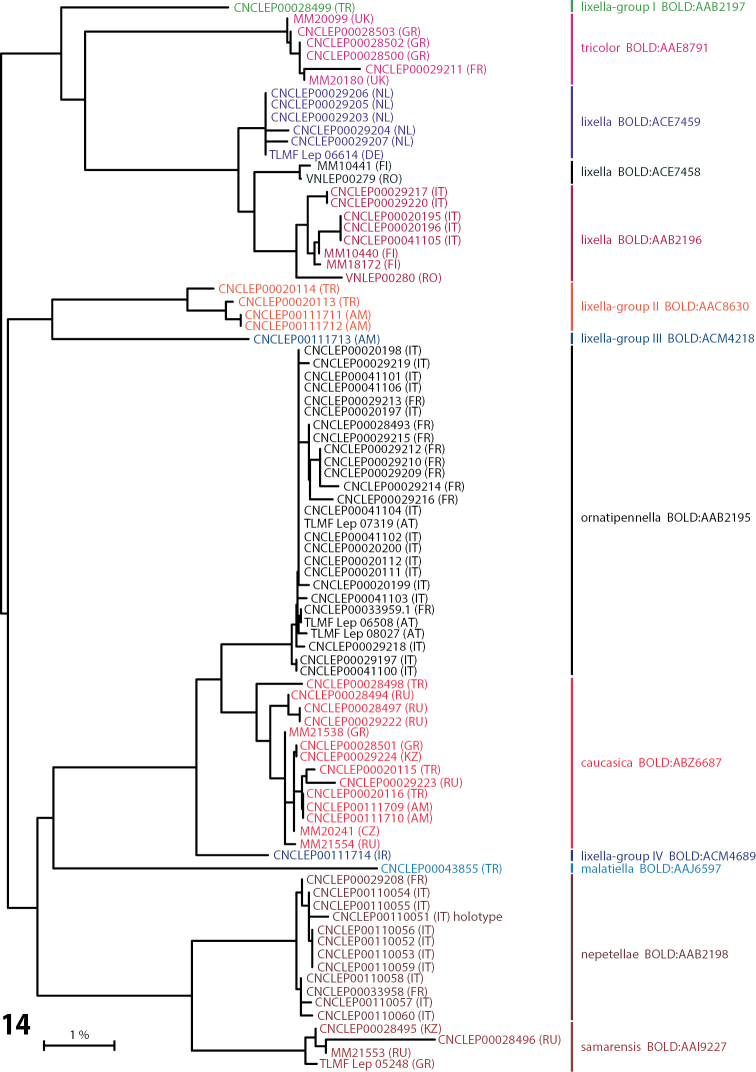
Neighbor-joining tree of K2P distances for the barcode region of the cytochrome *c* oxidase I gene among 87 specimens representing 13 species clusters of the *Coleophora lixella* group. End-branch labels are specimen ids followed by the geographic area in parentheses and the Barcode Index Number (BIN). Scale bar = 1%.

The majority of BINs had low intra-group divergence and thus seemed taxonomically well defined, including the new species, *Coleophora nepetellae*. This is congruent with the relatively subtle but consistent differences observed in genitalia which have been used by authors to distinguish those species. However, some specimens in four BINs (BOLD:AAB2197, BOLD:AAC8630, BOLD:ACM4218, BOLD:ACM4689, also labelled as ‘*lixella*-group I–IV in Fig. 14) were characterized by pronounced barcode divergence similar or exceeding those of described species, suggesting that they constituted putative undescribed diversity, as has been documented elsewhere in Lepidoptera (for examples, see Huemer and Hebert 2011, Huemer et al. 2012, Huemer et al. 2013, Kaila and Mutanen 2012, Landry and Hebert 2013, Mutanen et al. 2012a, b, Segerer et al. 2011, Wilson et al. 2010). Additionally, specimens identified as *Coleophora lixella* on the basis of genitalia separated into three different BINs which showed the shortest inter-group distances (1.4–1.6%) among all species analyzed. These *Coleophora lixella* specimens were all from continental Europe. Retrospectively we observed that the three *Coleophora lixella* BINs correlated with minor differences in genitalia among them but our sampling was too limited to clarify the situation. This suggests either marked haplotype variation within this species, or cryptic diversity. Further study is needed using more extensive material, but resolution of this problem is beyond the scope of the present paper.

**Table 1.** Percent sequence divergence in cytochrome *c* oxidase I gene among 87 specimens representing 13 species clusters (BINs) of the *Coleophora lixella* group. Cells below diagonal = mean inter-cluster distances in %; diagonal cells = mean intra-cluster distances. “BOLD:ABC1234” = Barcode Index Numbers.

	lixella-group I BOLD:AAB2197	tricolor BOLD:AAE8791	lixella BOLD:ACE7459	lixella BOLD:ACE7458	lixella BOLD:AAB2196	lixella-group II BOLD:AAC8630	lixella-group III BOLD:ACM4218	ornatipennella BOLD:AAB2195	caucasica BOLD:ABZ6687	lixella-group IV BOLD:ACM4689	malatiella BOLD:AAJ6597	nepetellae BOLD:AAB2198	samarensis BOLD:AAI9227
lixella-group I BOLD:AAB2197 (n=1)	n/c												
tricolor BOLD:AAE8791 (n=6)	7.0	0.8											
lixella BOLD:ACE7459 (n=6)	4.7	6.3	0.3										
lixella BOLD:ACE7458 (n=2)	5.5	6.4	1.6	0.2									
lixella BOLD:AAB2196 (n=8)	5.1	6.7	1.4	1.4	0.5								
lixella-group II BOLD:AAC8630 (n=4)	5.8	9.5	7.4	8.3	7.9	0.6							
lixella-group III BOLD:ACM4218 (n=1)	6.8	8.3	6.8	7.5	7.6	5.4	n/c						
ornatipennella BOLD:AAB2195 (n=27)	7.1	9.4	7.6	8.3	7.9	7.3	7.4	0.2					
caucasica BOLD:ABZ6687 (n=14)	9.1	10.2	9.7	10.1	10.0	8.9	8.9	3.5	0.7				
lixella-group IV BOLD:ACM4689 (n=1)	6.9	8.9	8.1	8.5	8.3	6.8	7.1	2.7	3.9	n/c			
malatiella BOLD:AAJ6597 (n=1)	7.5	8.9	7.6	7.9	7.7	8.4	8.6	8.2	9.6	7.4	n/c		
nepetellae BOLD:AAB2198 (n=12)	9.2	9.6	8.8	9.1	9.3	8.0	7.7	7.4	8.9	7.6	9.2	0.2	
samarensis BOLD:AAI9227 (n=4)	8.4	9.3	8.2	8.5	8.2	5.9	6.4	7.4	8.7	7.3	8.5	3.9	0.1

### New continental records of *Coleophora tricolor*

Barcode Index Number: BOLD:AAE8791

Barcoding results revealed that *Coleophora tricolor* occurs in southern France and in Greece (Fig. 14 and online dataset given above). This indicates that this species, previously thought to be restricted to Great Britain, has a much wider distributional range than previously considered. This also suggests that many existing continental records of *Coleophora lixella* or *Coleophora ornatipennella* that were not checked by genitalia dissections should be verified for their accuracy and for the possible misidentification of additional *Coleophora tricolor* specimens.

Our results also highlight the difficulty in recognizing the species of the *lixella* group from morphology alone. Differences in external aspect, if present, are subtle and may be blurred by slight variations and compounded by specimen wear. Illustrations of the genitalia of species of the *lixella* group in the literature are: *Coleophora caucasica* (Anikin 2001); *lixella*, *malatiella*, *nepetellae* (as “*lixella* cf *nevadella*”), *lixella*, *ornatipennella* (Nel 2001); *nevadella* (Baldizzone 1985); *malatiella* (Toll 1952). These illustrations differ greatly in quality and comparisons are problematic. Genitalia differences are also small and careful preparations are required to examine and compare them. These will be dealt with more comprehensively in a work in preparation on the *lixella* group by the first author. However, we give here the main differences between *Coleophora tricolor* and the pair *Coleophora lixella* – *Coleophora ornatipennella*, which most closely resemble each other.

Externally *Coleophora tricolor* can be tentatively distinguished from both *Coleophora lixella* and *Coleophora ornatipennella* by the fuscous annulations on the upperside of the distal half of the antenna, whereas the latter two species have that part of the antenna white, as do the other species of the *lixella* group. This character was pointed out by Emmet (1996: 276) but given its relative subtlety, it could be subject to geographic variation that has not been evaluated. Moreover, one must be careful to check the upper side of the antenna only: it is not uncommon for collection specimens to have the distal part of the antennae twisted around with the annulate lower sides turned upward.

In genitalia, males of *Coleophora tricolor* have the ventral edge of the valvula conical, and the apex of the sacculus angular and broad; in *Coleophora lixella* and *Coleophora ornatipennella*, the ventral edge of the valvula is broadly rounded, as is the apex of the sacculus; the cucullus is more pronouncedly upturned in *Coleophora lixella* than in the other two species. The appendix of the phallotheca has 13–14 coils in both *Coleophora tricolor* and *Coleophora ornatipennella*, versus only 10–11 in *Coleophora lixella*; in *Coleophora tricolor* the coils are wider in the middle of the appendix whereas in *Coleophora ornatipennella* they are gradually widened towards the apex.

Females of *Coleophora tricolor* have the colliculum about as long as the sterigma and the lateral bars of the sterigma with sharply delineated, concave outer edges and with apices outwardly curved; the spinules of the ductus bursae are small and restricted to the straight distal section except for a short patch at the anterior end of the first loop. Females of *Coleophora lixella* have a similarly proportioned colliculum/sterigma but the apex of the lateral bars are straight and the outer edges sinuate, and the spinules of the ductus bursae larger and extended around three-quarters of the loop. In *Coleophora ornatipennella* the colliculum is extremely narrow and more than 1.5x longer than the sterigma, the lateral bars of the sterigma are broader with diffusely delineated edges, and the spinules of the ductus bursae are much finer and extended only to about half of the loop.

**Record details.** 1 ♂: France, Provence-Alpes-Côte d’Azur, Hautes-Alpes, Les Laus, 6 km N Col d’Izoard, 1800 m, 30.VI.2003, C & FK Gielis leg., specimen CNCLEP00029211,genitalia slide MIC 6830, barcoded (coll. van der Wolf); 3 ♂: Greece, Macedonia, Kozani, near Xirolimni Village, 3.VI.2005, T. Nupponen leg., specimens CNCLEP00028500, 28502, 28503, genitalia slides MIC 5297, MIC 5299, barcoded (CNC).

## Supplementary Material

XML Treatment for Coleophora nepetellae
